# Comparison Between 24-2 ZEST and 24-2 ZEST FAST Strategies in Glaucoma and Ocular Hypertension Using a Fundus Perimeter

**DOI:** 10.1097/IJG.0000000000002358

**Published:** 2024-01-12

**Authors:** Dario Romano, Francesco Oddone, Giovanni Montesano, Paolo Fogagnolo, Benedetta Colizzi, Lucia Tanga, Sara Giammaria, Chiara Rui, Luca M. Rossetti

**Affiliations:** * Eye Clinic, ASST Santi Paolo e Carlo – San Paolo Hospital, University of Milan, Milan; † Glaucoma Unit, IRCCS Fondazione Bietti, Rome; ∥ CenterVue SpA, Padua, Italy; ‡ City, University of London–Optometry and Visual Sciences; § National Institute for Health Research (NIHR) Biomedical Research Centre at Moorfields Eye Hospital, NHS Foundation Trust and UCL Institute of Ophthalmology, London, UK

**Keywords:** glaucoma, ocular hypertension, visual field, fundus perimetry, fundus tracking

## Abstract

**Précis:**

Using a Compass (CMP) (CMP, Centervue, Padova, Italy) fundus perimeter, Zippy Estimation by Sequential Testing (ZEST) FAST strategy showed a significant reduction in examination time compared with ZEST, with good agreement in the quantification of perimetric damage.

**Purpose:**

The aim of this study was to compare the test duration of ZEST strategy with ZEST FAST and to evaluate the test-retest variability of ZEST FAST strategy on patients with glaucoma and ocular hypertension.

**Patients and Methods:**

This was a multicenter retrospective study. We analyzed 1 eye of 60 subjects: 30 glaucoma patients and 30 patients with ocular hypertension. For each eye we analyzed, 3 visual field examinations were performed with Compass 24-2 grid: 1 test performed with ZEST strategy and 2 tests performed with ZEST FAST. Mean examination time and mean sensitivity between the 2 strategies were computed. ZEST FAST test-retest variability was examined.

**Results:**

In the ocular hypertension cohort, test time was 223±29 seconds with ZEST FAST and 362±48 seconds with ZEST (38% reduction, *P*<0.001). In glaucoma patients, it was respectively 265±62 and 386±78 seconds (31% reduction using ZEST FAST, *P*<0.001). The difference in mean sensitivity between the 2 strategies was −0.24±1.30 dB for ocular hypertension and −0.14±1.08 dB for glaucoma. The mean difference in mean sensitivity between the first and the second test with ZEST FAST strategy was 0.2±0.8 dB for patients with ocular hypertension and 0.24±0.96 dB for glaucoma patients.

**Conclusions:**

ZEST FAST thresholding provides similar results to ZEST with a significantly reduced examination time.

Visual field (VF) examination is the clinical standard for the diagnosis and follow-up of glaucoma. A correct estimation of perimetric defects is a fundamental indicator to monitor change in glaucoma, with implications for clinical management.^1^ Many factors can affect the interpretation of a single or of a series of VF tests.^2,3^ In fact, strong cooperation is needed from patients to obtain a reliable assessment of VF damage. Patients are indeed required to maintain central fixation and to promptly respond to the presented stimuli for the entire duration of the exam. Variability in patient attention contributes to generating short-term and long-term fluctuations that make the detection of glaucoma progression challenging.^4^

In the Humphrey technology, the Swedish Interactive Threshold Algorithms (SITA) have enabled large reductions in examination time compared with the Full Threshold algorithm, for a long time considered the gold standard. SITA Standard reduces the duration of the test by up to 50%.^5^ SITA Fast examinations achieve even shorter durations^6^ with minimal difference in accuracy compared with Full Threshold algorithm.^7^ This is clinically meaningful because many studies demonstrated how perimetric sensitivity and variability is affected by increased examination duration. This effect is often attributed to fatigue and is influenced by stimulus eccentricity patient age and is more conspicuous in areas adjacent to field deficits.^8,9^ Reducing test time without compromising accuracy is therefore of paramount importance for clinical practice.^10^

Compass (CenterVue, Padova, Italy) is a fundus automated perimeter equipped with a scanning ophthalmoscope and eye tracker which compensates for eye movements reducing the effect of fixation instability.^11,12^ The device also provides high-quality color photographs of the optic nerve and of the central retina, allowing direct comparison of structural features and VF sensitivity.^13^ The default thresholding strategy in Compass (CMP) is the Zippy Estimation by Sequential Testing (ZEST),^14^ an adaptive Bayesian method.^15^ CMP ZEST showed good agreement with SITA Standard, with a difference similar to that reported by Wild et al^16^ between the Humphrey SITA standard and Humphrey Full Threshold in and within clinically acceptable limits. The average examination time of ZEST is nearly halved compared with a 4-2 staircase strategy, previously used by the device and similar to a Full Threshold strategy, but it is still longer compared with the commonly used SITA Fast.

In 2022, Turpin and McKendrick^17^ introduced ARBON (Artificial Responses Based on Neighbors), an algorithm that has provided in a computer-simulated perimetry a reliable threshold estimation with a reduced number of presented stimuli. With the implementation of ARBON, a new CMP strategy called ZEST FAST has been developed, with the aim of reducing test time, without compromising accuracy.

This is the first clinical study with an ARBON-implemented strategy, and our objective is to retrospectively evaluate the ZEST FAST strategy and compare it with ZEST.

## MATERIALS AND METHODS

This multicenter retrospective study was conducted at the Eye Clinic of San Paolo Hospital, ASST Santi Paolo e Carlo, Milan, and at the IRCCS G.B. Bietti Foundation, Rome. The protocol was approved by the institutional review board (Comitato Etico Milano Area 1, No. 0034559, July 31, 2023) and carried out in accordance with the tenets of the Declaration of Helsinki.

### Study Population

We analyzed VF examinations performed by ocular hypertension (OHT) and glaucoma patients between January and December 2022 in our Glaucoma Unit. Glaucoma definition was based on VF damage (a cluster of three or more contiguous abnormal locations on pattern deviation map, 24-2 CMP ZEST strategy) with corresponding typical changes of the optic nerve head and/or ganglion cell layer (GCL) evaluated by means of SD-OCT, irrespective of IOP. For the OHT group, we considered patients with normal VF (24-2), normal optic nerve head and GCL (SD-OCT) and baseline intraocular pressure >21 mm Hg without treatment. Inclusion criteria were age of 18 years or more; spherical refractive error ranging between −10 diopters (D) and +6 D, with maximum ±2 D of astigmatism; best-corrected visual acuity >0.6; no history of ocular trauma. We excluded from the analysis patients with known systemic diseases, ocular diseases other than glaucoma and OH, patients using drugs that could affect the VF examination, or those who had received ocular surgery (except uncomplicated cataract surgery) <6 months before the first study examination.

VF tests were performed in both eyes, but only 1 eye per patient was chosen at random to be analyzed. In the case of unilateral glaucoma, only the affected eye was analyzed.

### Study Examinations

During routine visits in our Glaucoma Unit, patients who have already been tested with CMP, 24-2 ZEST, performed the examination with same grid and strategy for a correct comparison with previous exams. If the VF were considered stable, to obtain a future time saving and a more comfortable experience for the patient, the VF was tested again with the same grid but ZEST FAST strategy. The examination was then repeated with the same strategy to get a reliable baseline for the upcoming visits, as recommended by the European Glaucoma Society when switching from a perimetric strategy to another.^18^ To minimize the fatigue effect, for each patient, rest breaks were carried out between each test.

We included in this analysis subjects who had already performed 3 VF examinations during the same visit: 1 test with ZEST strategy and 2 tests with ZETS FAST. In all cases, the test was a 24-2 pattern, 200-millisecond stimulus duration, Goldmann III stimulus size, retinal tracking ON, and measurement of foveal location ON. 3We only included in this analysis patients able to perform all 3 reliable tests (false positive <18% and false negative <30%).

### Study Outcomes

The primary end point was to test a reduction of the average examination time of 30% or more with ZEST FAST compared with ZEST, which is consistent to the reduction found between SITA Standard and SITA Fast.^19^ Secondary outcomes were the quantification of the difference in Group Mean Sensitivity (GMS), defined as the average of per-test mean sensitivity values across all subjects in the same group (glaucoma or OHT) and across all stimulus locations; the agreement between pointwise threshold values between ZEST and ZEST FAST, quantified with their 95% limits of agreement (LoA), and the test-retest variability for ZEST FAST, quantified with its 95% limits of repeatability. LoA and limits of repeatability were calculated using Bland-Altman analysis.^20^ Another analysis was made to describe differences across various test locations, computing the mean of the within-subject differences in sensitivity between ZEST and ZEST FAST for each of the 52 tested locations. Differences in examination time, GMS, false positive and false negative rates were tested using a paired-samples *t* test.

All analyses were done using Matlab version R 2023a (The Mathworks Inc.).

## RESULTS

A total of 60 subjects were included: 30 subjects with ocular and 30 subjects with glaucoma. The mean deviation calculated on the 24-2 ZEST grid was −0.27±1.36 dB for OHT subjects and −5.95±5.56 dB for glaucomatous patients. Table 1 reports the study population across the 2 groups.

**TABLE 1:** Study Population

				Sex		Eyes	
Group	Subjects N	Age	Mean deviation	Male, N (%)	Female, N (%)	Right, N (%)	Left, N (%)
Ocular hypertension	30	50.6±13.9	−0.27±1.36 dB	9 (30)	21 (70)	21 (70)	9 (30)
Glaucoma	30	66.1±11.4	−5.95±5.56 dB	13 (43)	17 (57)	19 (63)	11 (37)

The Group Mean Examination Time was computed for ZEST and ZEST FAST (Table 2). The average reduction of the examination time with ZEST FAST was 38% in the OHT group and 31% in the glaucoma group (*P*<0.001 for both groups).

**TABLE 2:** Comparison Between Group Mean Examination Time (GMET) [seconds (s)] for ZEST and ZEST FAST in Ocular Hypertensive and Glaucoma Patients

Group	Subjects N	GMET ZEST (s)	GMET ZEST FAST (s)	Reduction (%)	*P*
Ocular hypertension	30	362±48	223±29	38	<0.001
Glaucoma	30	386±78	265±62	31	<0.001

ZEST indicates Zippy Estimation by Sequential Testing.

The GMS and Sensitivity Standard Deviation were computed for both study groups and thresholding algorithms. All grid points were used for the calculation, with the exclusion of the 2 locations at the optic disc and the fovea (Table 3). The difference in GMMS between the 2 methods (ZEST − ZEST FAST) was −0.24±1.30 dB (−5.15, 2.44) and −0.14±1.08 (−2.94, 2.67) dB for OHT and glaucoma patients respectively (Table 4).

**TABLE 3:** Comparison Between Group Mean of the Mean Sensitivity (GMMS) [decibels (db)] for ZEST and ZEST FAST in Ocular Hypertensive and Glaucoma Patients

Group	Subjects N	GMMS ZEST (dB)	GMMS ZEST FAST (dB)	*P*
Ocular hypertension	30	27.73±1.51	27.97±0.91	0.421
Glaucoma	30	21.30±5.44	21.44±5.22	0.907

ZEST indicates Zippy Estimation by Sequential Testing.

**TABLE 4:** Comparison of Group Mean of the Mean Sensitivity Differences (GMMSD) [decibels (db)] and Group Mean of the Mean Absolute Differences (GMMASD) [decibels (db)] Between First and Second Repetition of ZEST FAST and Between ZEST and ZEST FAST

		ZEST FAST − ZEST FAST		ZEST − ZEST FAST		*P*	
Group	Subjects N	GMMSD (dB)	GMMASD (dB)	GMMSD (dB)	GMMASD (dB)	MSD	MASD
Ocular hypertension	30	0.20±0.80	1.79±0.43	−0.24±1.30	2.11±0.74	0.864	0.032
Glaucoma	30	0.40±0.97	2.80±1.09	−0.14±1.08	3.03±1.18	0.927	0.365

A paired *t* test is used to compare values between first and second repetition of ZEST FAST with those between ZEST and ZEST FAST, for both Mean Sensitivity Differences (MSD) and Mean Absolute Sensitivity Differences (MASD).

MASD indicates mean absolute sensitivity differences; MSD, mean sensitivity differences; ZEST, Zippy Estimation by Sequential Testing.

An analysis was made to evaluate differences of the mean sensitivity (MS) within-subjects and pointwise sensitivity differences across all test locations, between both ZEST FAST tests (test and retest) and between ZEST and ZEST FAST tests. The group mean of the mean sensitivity differences (GMMSD) and one SD (SSDD) between the 2 ZEST FAST repetitions and between ZEST and the 2 ZEST FAST are reported in Table 4. The same calculations are reported considering the GMMSD.

**FIGURE 1:**
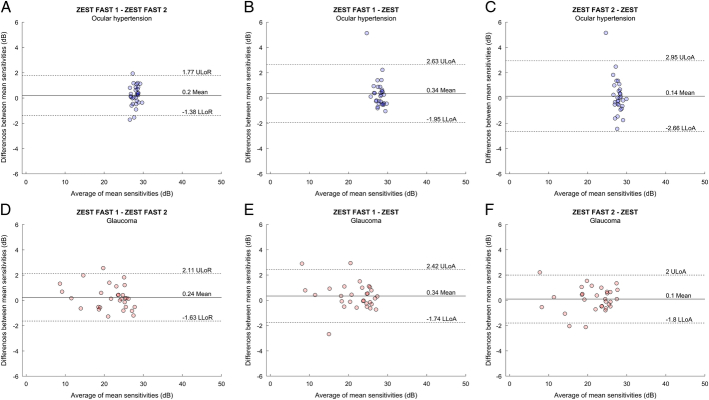
Bland-Altman plots for MS for ocular hypertension subjects (blue dots) and for glaucoma patients (red dots). The plots are depicted for all the combinations between ZEST FAST 1 − ZEST FAST 2 (A, D, respectively), ZEST FAST 1 − ZEST (B, E) and ZEST FAST 2 − ZEST (C, F). The area between the 2 dotted lines indicates the 95% limits of agreement on the test-retest difference. The black solid line indicates the mean difference between test-retest MS measurements. LLoA indicates lower limits of agreement; LLoR, lower limits of repeatability; MS, mean sensitivity; ULoA, upper limits of agreement; ULoR, upper limits of repeatability; ZEST, Zippy Estimation by Sequential Testing.

**FIGURE 2:**
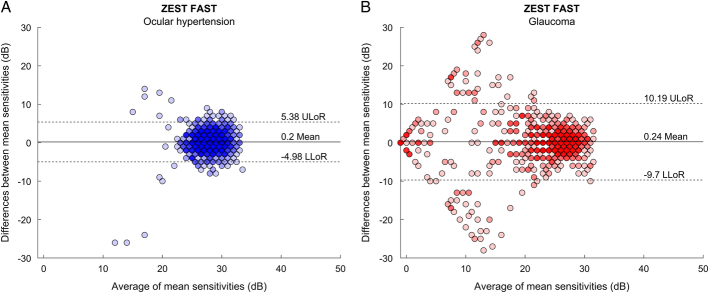
Bland-Altman plots for all pointwise sensitivity measurements from Compass ZEST FAST for ocular hypertension subjects (A) and for glaucoma patients (B). The area between the 2 dotted lines indicates the 95% limits of agreement on the test-retest difference. The black solid line indicates the mean difference between test-retest point-wise sensitivity measurements. LLoR indicates lower limits of repeatability; ULoR, upper limits of repeatability; ZEST, Zippy Estimation by Sequential Testing.

We used Bland-Altman plots to study MS and all pointwise sensitivities of test-retest values of ZEST FAST. The mean difference in MS between the first and the second test with ZEST FAST strategy was 0.2±0.8 dB for OHT subjects and 0.24±0.96 dB for glaucoma patients. The 95% LoA for MS are depicted in Figure 1. They were 31% narrower for ZEST FAST 1 − ZEST FAST 2 (LoA: −1.38, 1.77 dB) compared with ZEST FAST 1 − ZEST (LoA: −1.95, 2.63 dB) and 44% narrower compared with ZEST FAST 2 − ZEST (LoA: −2.66, 2.95−dB) in the OHT subjects (Figs. 1A–C). The 95% LoA were 10% narrower for ZEST FAST 1 − ZEST FAST 2 (LoA: −1.63, 2.11 dB) compared with ZEST FAST 1 − ZEST (LoA: −1.74, 2.42 dB) and 2% narrower compared with ZEST FAST 2 − ZEST (LoA: −1.8, 2.0 dB) in the glaucoma patients (Figs. 1D–F). The mean test-retest difference in the pointwise sensitivities measurements was −0.2±2.64 dB for OHT subjects and 0.24±5.07 dB for glaucoma patients. Bland-Altman plots for all sensitivities are reported in Figure 2. For the pointwise sensitivities measurements, the 95% LoA is narrower, as expected, for OHT and for glaucoma above 20 dB.

**FIGURE 3:**
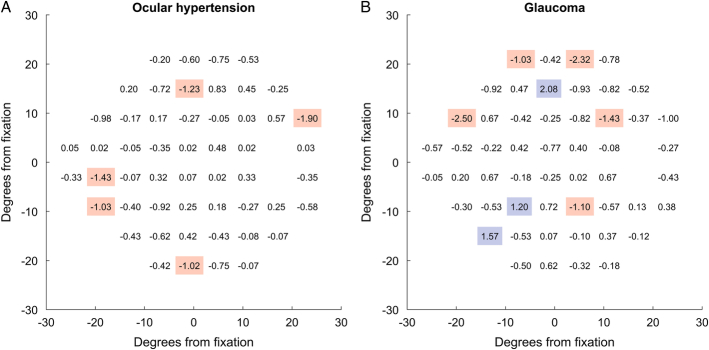
Pointwise within-subject differences in sensitivity [decibels (db)] for each of the 52 locations of the 24-2 grid, between Zippy Estimation by Sequential Testing (ZEST) and ZEST FAST tests, for ocular hypertension subjects (A) and for glaucoma patients (B). Differences below −1 dB are highlighted in orange while differences above +1 dB are highlighted in blue.

The mean of the pointwise within-subject differences in sensitivity between ZEST and ZEST FAST are reported in Figure 3 for OHT subjects and glaucoma patients, respectively. There were only 5 locations for ocular hypertensive subjects and 8 locations for glaucomatous patients exceeding 1 dB in absolute difference between the 2 strategies.

The average percentage of false positives was lower for ZEST FAST compared with ZEST in both OHT subjects, 0.5% and 0.9%, respectively (*P*=0.535), and glaucomatous patients, 0.9% and 2.3%, respectively (*P*=0.663). We did not find a statistically significant difference for false negatives too, with a lower average percentage in ZEST FAST compared with ZEST in the OHT group, 2.6%, and 4.6%, respectively (*P*=0.192), and 7.9% in ZEST FAST compared with 6% in ZEST (*P*=0.368) for glaucomatous patients.

## DISCUSSION AND CONCLUSIONS

This study compared the CMP ZEST and ZEST FAST thresholding strategies in a cohort of OHT and glaucoma patients.

We found a significant difference in the average examination time between ZEST and ZEST FAST, which was reduced by 38% in subjects without glaucoma (OH) and 31% in patients with glaucomatous damage (glaucoma group). This is consistent with the reduction in the examination time found comparing SITA Fast with SITA Standard for normal visual subjects. In contrast, the time gain for glaucomatous patients seemed smaller than that reported for SITA Fast. The absolute examination time seems to be slightly longer with ZEST FAST than with SITA Fast for both normal and glaucomatous visual fields.^19^ It should be considered that our study not only used different testing strategies but also different devices (Compass instead of HFA). Notably, fundus tracking, while increasing projection accuracy, might also increase test time if there are gaps in the acquisition of a fundus image, which would temporarily halt the test.

Test performance, as measured by false positive rates, seemed to be better for ZEST FAST compared with ZEST in both OHT subjects, 0.5% and 0.9%, respectively, and glaucomatous patients, 0.9% and 2.3%, respectively, which might indicate an improvement in test performance with shorter examination time. We found a lower average percentage of false negatives in ZEST FAST compared with ZEST in the OHT group but a higher rate in the glaucoma group, 2.6% and 4.6%, respectively, which can be explained by the increased variability in threshold values found in glaucomatous eyes and worsened by the fatigue effect for repeated examinations.^21^ It should also be noted that test duration also affects the accuracy with which false responses are estimated. Anyway, these results did not show statistically significant differences, which could be due to high variability in a relatively small sample or to the lack of real differences. Further investigations are needed to assess this issue.

The ZEST FAST group mean MS was higher than ZEST for both normal and glaucomatous visual fields, but the absolute differences were inferior to 1 dB, which is consistent with our prespecified limits. The difference in group mean of the mean sensitivity between the 2 strategies in the glaucoma group was 0.14 dB, which is also inferior (in absolute magnitude) to 0.9 dB, previously reported between 4-2 and ZEST strategies.^22^ SITA Fast has also been shown to measure higher group mean MS compared with SITA Standard in a cohort of glaucomatous patients, and the difference (0.9 dB) was considerably greater than the one found between ZEST FAST and ZEST.^23^ This significant difference could be explained by differences in threshold acquisition strategies and by the presence of fundus tracking in CMP. This different approach could lead to a more precise threshold estimation and lower test-retest variability in the MS but with a longer examination time for CMP compared with HFA.

Group mean sensitivity and standard deviations obtained with ZEST FAST are comparable to those obtained with ZEST on the same patients, suggesting that the 2 strategies provide similar estimates of the VF.

The 95% LoA for Mean Sensitivity values and Pointwise thresholds for ZEST test-retest are comparable and in line with those presented for comparison between CMP ZEST and HFA SITA standard strategies by Montesano and colleagues. For OHT patients, LoA for MS for ZEST FAST (−1.38 dB; 1.77 dB) are comparable to those found in healthy subjects for ZEST (−1.31 dB; 1.63 dB) and narrower compared with HFA SITA Standard (−2.84 dB; 2.91 dB). For glaucoma patients, ZEST FAST appears more repeatable, with LoAs −1.63 dB; 2.11 dB, narrower than what was previously reported for both CMP ZEST (−2.26 dB; 3.14 dB) and HFA SITA Standard (−3.11 dB; 3.11 dB).^12^ Of course, these comparisons are limited by the fact that these data were collected in different cohorts of patients.

Thanks to rest breaks allowed between each test, fatigue effect was limited, and we have not found any significant difference between the first and second test with ZEST FAST concerning both examination duration and mean sensitivities (Table 4).

One limitation of our study is the small sample size. Test-retest threshold distributions for ZEST FAST of Glaucoma patients (Supplementary Fig. 1, Supplemental Digital Content 1, http://links.lww.com/IJG/A870) do not cover all sensitivity values, which makes it difficult to compare pointwise test-retest variability with previously published data.^24^

Another limitation is the retrospective design of the study, which prevented us from analyzing data from completely healthy subjects. However, this also provided us with data derived from a clinically relevant scenario, avoiding hyperselection of study participants.^25^ Another limitation related to the design of the study concerns the sequence of VF examinations, which was not randomized. Each subject performed the first test with ZEST strategy and then 2 consecutive tests with ZEST FAST strategy: a better performance during tests with ZEST FAST could be due to the learning effect. However, this could also be counteracted by worsening the performance due to fatigue effect.^23^ In general, however, these patients were not naïve to perimetry. Glaucoma patients were not stratified according to disease severity. This could have resulted in an uneven representation of data, but even with a shortage of advanced defects, the range of VF damage was sufficiently large to allow for a reliable evaluation across the whole spectrum of glaucoma damage.

ARBON is an algorithm that has provided a reliable threshold estimation with a reduced number of presented stimuli in computer-simulated perimetry. Implementing information from initially tested loci, ARBON can provide a faster threshold estimation of neighbors’ locations. This method can be added to a perimetry test procedure to speed up the examination without compromising the returned sensitivity and without altering any parameter of the visual field-testing strategy. To the best of our knowledge, ZEST FAST is the first visual field strategy implementing the ARBON algorithm, which has led to a reduction in the number of presentations without altering the testing logics and parameters. Since Turpin and McKendrick have validated this algorithm through computer-simulated perimetry testing, this is the first clinical study with an ARBON-implemented strategy.

In conclusion, these data suggest that ZEST FAST thresholding is comparable to ZEST strategy in all aspects, including test-retest variability, with a significantly reduced examination time.

## Supplementary Material
