# Supplementary figures and images for: Comparison Between 24-2 ZEST and 24-2 ZEST FAST Strategies in Glaucoma and Ocular Hypertension Using a Fundus Perimeter
Source: J Glaucoma. 2024 Jan 12;33(3):162–7. doi: 10.1097/IJG.0000000000002358 (PMC10901223; doi:10.1097/IJG.0000000000002358)

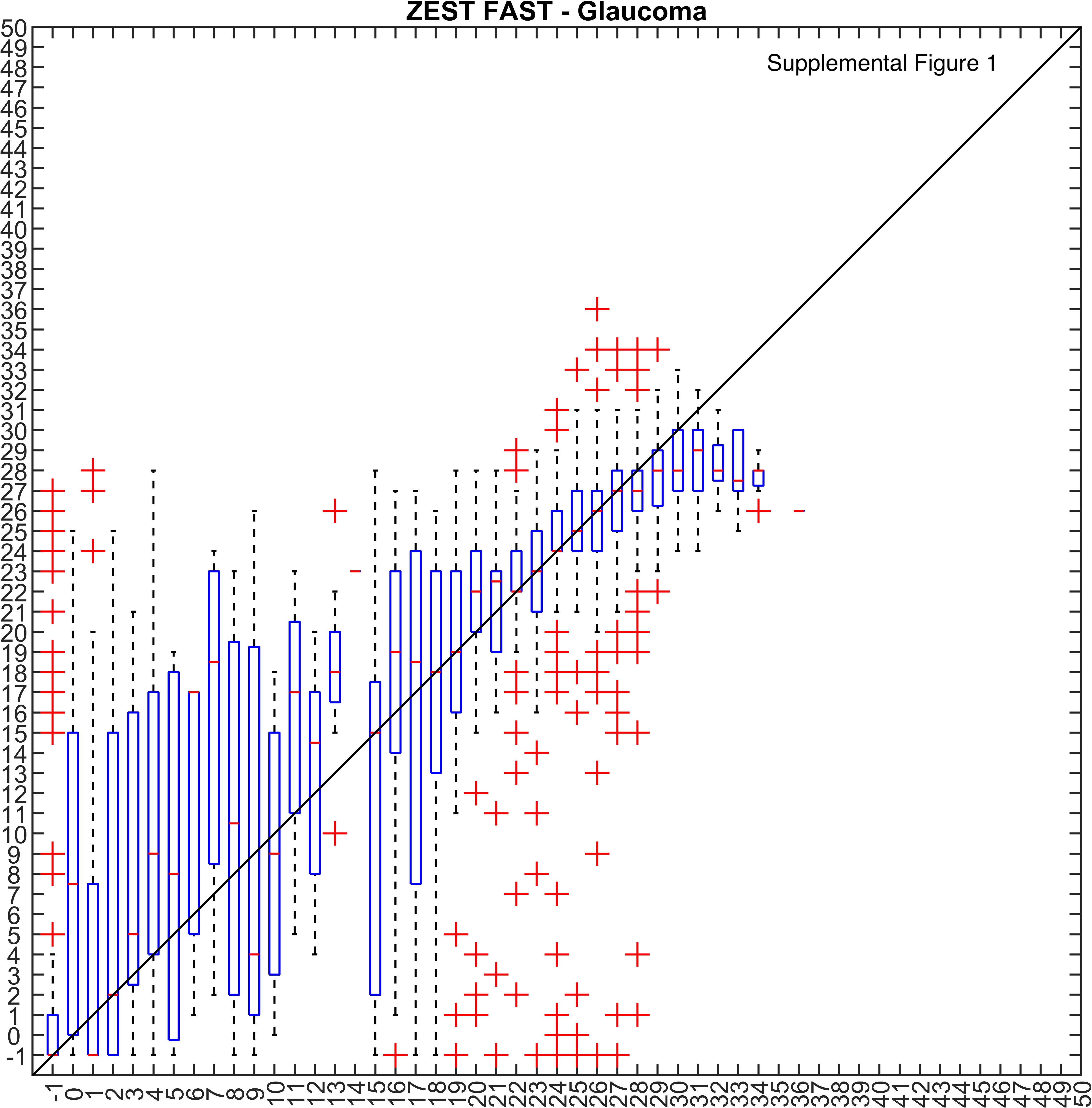

Supplement: Supplementary file 1 [file ijg-33-162-s001.jpg]
